# Severe Cholestasis in Neonates with Hemolytic Disease of the Fetus and Newborn—A Case Report

**DOI:** 10.3390/jcm13051272

**Published:** 2024-02-23

**Authors:** Agnieszka Drozdowska-Szymczak, Julia Proczka, Natalia Mazanowska, Artur Ludwin, Paweł Krajewski

**Affiliations:** 1 Department of Neonatology and Neonatal Intensive Care, Institute of Mother and Child, Kasprzaka 17a, 01-211 Warsaw, Poland; 2 Department of Obstetrics and Gynecology, Institute of Mother and Child, Kasprzaka 17a, 01-211 Warsaw, Poland; 3 Department of Obstetrics and Gynecology, Medical University of Warsaw, Pl. Starynkiewicza 1/3, 02-015 Warsaw, Poland

**Keywords:** neonate, cholestasis, hemolytic disease of the fetus and newborn, intrauterine blood transfusion, iron overload, conjugated hyperbilirubinemia

## Abstract

Hemolytic disease of the fetus and newborn (HDFN) may cause severe cholestasis with direct bilirubin concentrations reaching up to 50 times the upper limit of normal. This case report describes twins whose highest direct bilirubin concentrations were 32.2 mg/dL and 50.2 mg/dL, with no significant signs of hepatic impairment. The index pregnancy was complicated by Rhesus factor immunization with anti-D antibodies present in maternal serum, which caused fetal anemia requiring intrauterine blood transfusions. Complementary tests demonstrated Rhesus D alloimmunization as the sole cause of cholestasis. To the best of our knowledge, this is the first study to describe such elevated direct bilirubin concentrations caused by HDFN.

## 1. Introduction

Hemolytic disease of the fetus and newborn (HDFN) is an immune-mediated disorder caused by the mother’s antibodies directed against fetal red blood cells (RBCs). Alloimmunization can be caused by any Rhesus antigen (D, d, C, c, E, e) or other antigens absent in maternal RBCs but inherited from the father. Fetal blood cells may migrate into maternal circulation and stimulate the production of immunoglobulin G (IgG) that can cross the placenta. Maternal IgG antibodies attach to fetal RBCs, causing hemolysis leading to anemia and increased bilirubin production.

Bilirubin is an end-product of heme degradation, primarily deriving from hemoglobin. Serum transport of poorly water-soluble bilirubin requires binding with albumin as a carrier. This indirect (unconjugated) bilirubin can cross the blood–brain barrier (BBB) and exert a neurotoxic effect. 

Unconjugated bilirubin is transported to the liver, where it is modified into a water-soluble compound—direct (conjugated with glucuronic acid) bilirubin. This form cannot cross the BBB and does not affect the brain. Direct bilirubin is actively excreted into the bile and to the small intestine, where it is transformed into bile pigments (stercobilin) by bacterial enzymes.

Hyperbilirubinemia is not a concern when considering fetal life, owing to placental clearance. After birth, the neonatal liver cannot metabolize bilirubin due to the immaturity of its enzyme activity. This results in unconjugated hyperbilirubinemia, which may require treatment to prevent kernicterus and encephalopathy.

During alloimmunized pregnancies, intrauterine blood transfusions (IUTs) may be necessary to treat fetal anemia. To assess the need for intrauterine therapy, the middle cerebral artery peak systolic velocity (MCA PSV) values, expressed as multiples of the median (MoM), are used, with a value of MoM MCA PSV higher than 1.5 MoM regarded as an indication for cordocentesis and intrauterine transfusion [1].

IUT is often a life-saving procedure; however, its negative consequences should also be noted. Procedure-related complications may include fetal demise, acute fetal distress, volume overload with heart failure, chorioamnionitis, preterm rupture of membranes, or preterm labor. Suppression of fetal erythropoiesis, minimal risk for anaphylactic reactions, and transmission of viral diseases have also been observed [2]. 

Although HDFN is a rare cause of cholestasis, elevated direct bilirubin concentration is common [3,4]. It is caused mainly by iron overload of the liver, and is more frequent in neonates requiring IUTs, especially neonates experiencing Rhesus D alloimmunization [4].

The term cholestasis describes an interruption of bile flow or excretory hepatocyte dysfunction. Consequently, we have observed elevations of direct bilirubin, bile acids, and other bile component levels. The diagnosis is based on a direct bilirubin concentration exceeding 1 mg/dL (17.1 µmol/L) regardless of the total serum bilirubin (TSB) concentration [5]. Symptoms of cholestasis include jaundice, hepatomegaly, pale stools, and rarely, green discoloration of the skin [6]. Increased concentrations of conjugated bilirubin, gamma-glutamyl transpeptidase (GGT), transaminases, and alkaline phosphatase (ALP) are also often observed in patients with cholestasis. ALP alone should not be utilized as a diagnostic parameter because elevated levels of bone-specific alkaline phosphatase (BAP) are typical during the neonatal period [6].

## 2. Case Presentation

We present the case of male monochorionic diamniotic (MCDA) twins born at 34 weeks of gestation (para 5, gravida 5) via emergency cesarean section in the first stage of labor because of the transverse lie of the first twin. The index pregnancy was complicated by red cell alloimmunization with an anti-D antibody titer > 1:2000 and an anti-C antibody titer of 1:64, diet-controlled gestational diabetes mellitus, and hypertension. A single course of steroids (betamethasone) was given antenatally. The presence of fetal anemia required two IUTs for both fetuses, at 31 and 33 weeks of gestation. Before the first IUT, hemoglobin concentration levels of 9.1 g/dL in twin A and 8.9 g/dL in twin B were observed. Further details can be found in Table 1. 

Physical examination of the patients revealed subcutaneous edema, hepatosplenomegaly, and green skin discoloration. Twin A, weighing 3100 g, was delivered non-vigorous, with Apgar scores of 1, 4, 6, and 6 at 1, 3, 5, and 10 min after birth, respectively, an arterial cord blood pH of 7.07, and a base excess (BE) of −10.1 mmol/L. Twin B, weighing 3200 g, was delivered non-vigorous, with Apgar scores of 2, 4, 6, and 7 at 1, 3, 5, and 10 min after birth, respectively, an arterial cord blood pH of 7.11, and a BE of −9.0 mmol/L. 

Severe hyperbilirubinemia was observed in both neonates due to HDFN. The patients’ serious condition was considered as a contraindication for exchange transfusions on the first day of life. The newborns were treated with intensive phototherapy for four days and intravenous immunoglobulin (IVIG) infusion. 

**Figure 1:**
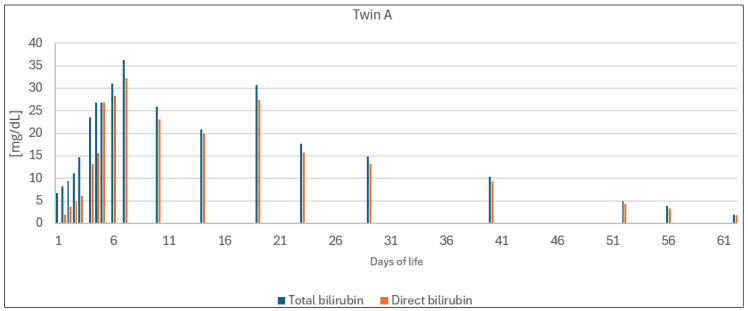
Bilirubin concentrations of twin A.

**Figure 2:**
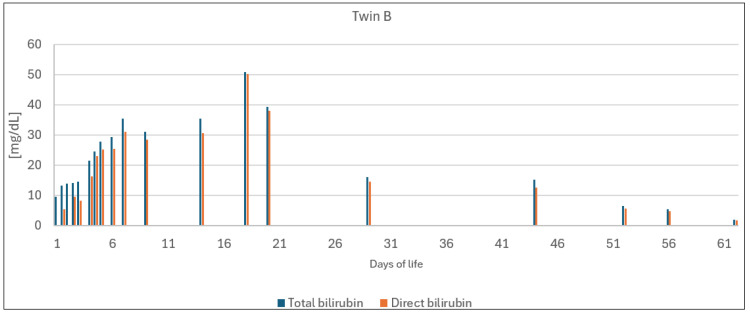
Bilirubin concentrations of twin B.

During the first weeks of hospitalization, we observed increasing bilirubin levels in both twins, with direct bilirubin making up most of their TSBs since their fourth day of life. Consequently, an exchange transfusion was not performed. In the following days, a gradual decrease in TSB concentrations was noted, and the twins were discharged with bilirubin levels vaguely exceeding the upper limit of normal. Their bilirubin values are presented in Figure 1 and Figure 2. Other treatment details can be found in Table 2.

Laboratory tests revealed severe cholestasis in both patients, with elevated GGT and transaminases. Their most extreme laboratory results are presented in Table 3. 

During hospitalization, laboratory value improvement was observed for both patients. The twins did not present coagulation disorders, and there was no need for albumin administration. Due to the suboptimal 25-hydroxyvitamin D status of twin A, high-dose vitamin D supplementation was administered, and twin B maintained adequate vitamin D levels during regular-dose supplementation.

Both patients temporarily passed pale stools, yet normal stool color was observed at discharge. Symptomatic treatments with ursodeoxycholic acid (UDCA), fat-soluble vitamins (A, D, E, K), docosahexaenoic acid (DHA), and formulas with medium chain triglycerides (MCT) were used.

Complementary tests were performed to rule out any causes of cholestasis other than HDFN. Serum alpha-1 antitrypsin levels were deemed normal. Thyroid stimulating hormone (TSH) levels and the activity of galactose-1-phosphate uridyl transferase (GALT) in RBCs were within normal range. TANDEM MS, GC–MS, and cystic fibrosis screening tests showed no abnormalities. No signs of Alagille syndrome were observed. Cortisol levels were within normal range. PCR-based panels for viruses were negative, and congenital cytomegalovirus infection and congenital toxoplasmosis were excluded.

Both twins required blood transfusions due to anemia. Twin A received three transfusions, while twin B received two transfusions.

Echocardiography on the first day of life revealed persistent pulmonary hypertension of the newborn (PPHN) and increased chamber volumes in both twins, with normal cardiac contractibility during vasopressor administration. In ensuing examinations, gradual normalization was observed.

Both patients required mechanical ventilation, nitrous oxide due to PPHN, and surfactant therapy due to respiratory distress on the first day of life. Catecholamines were administered due to circulatory failure. In both twins we also observed hypoglycemia on the first day of life, thrombocytopenia in the first week of life, and necrotizing enterocolitis (NEC). Parenteral nutrition with fish oil-based lipid emulsions was used during hospitalization. 

Both twins required antibiotic therapy. Potentially ototoxic ampicillin and gentamicin were used until the exclusion of early-onset sepsis (EOS) in the first four days of life. During their hospital stay, each twin also received gentamicin and vancomycin, as NEC treatment (twin A), and during late-onset sepsis before an antibiogram could be obtained (twin B).

Abdominal ultrasounds showed hepatosplenomegaly in both twins, with no abnormalities of the gall bladder or bile ducts. Cranial ultrasounds revealed abnormal hemodynamic parameters in cerebral vessels (reverse flow in the middle cerebral artery), increased periventricular echogenicity, and hyperechogenicity in the left thalamus of both patients. Signs of grade 1 intraventricular hemorrhage (IVH) were also noted: bilaterally in twin A, and on the right side in twin B. Both boys underwent brain magnetic resonance imaging (MRI), which revealed no significant abnormalities. Ophthalmic consultations were obtained multiple times for both twins; stage 2 retinopathy of prematurity was diagnosed, with no indication for treatment. Additionally, yellow discoloration of the retina was present.

Currently, the twins are 3.5 years old and do not require medication. They receive physical therapy and orofacial myofunctional therapy. There are no signs of cerebral palsy or neurodevelopmental impairment. Neurological examinations show no significant abnormalities. Although both patients receive audiological care due to suspected partial hearing loss, they do not require hearing aids.

## 3. Discussion

The severity of HDFN differs between mild anemia with hyperbilirubinemia and intrauterine fetal demise as a result of fetal hydrops. Anemia requiring multiple transfusions and hyperbilirubinemia with jaundice are often observed. Other postnatal complications include thrombocytopenia, iron overload, cholestasis, bilirubin-induced neurologic dysfunction, and kernicterus [3]. Long-term consequences of severe HDFN may consist of altered cardiovascular development, cerebral palsy, severe developmental delay, bilateral deafness, and blindness [7]. 

In the described case, both twins required two IUTs due to fetal anemia. These procedures were performed with no complications. No signs of hydrops fetalis were observed. During the neonatal period, both patients needed blood transfusions due to anemia. Twin A received three transfusions, while twin B received two transfusions

Hyperbilirubinemia caused by HDFN may require phototherapy or exchange transfusions [8,9,10,11,12]. The latter is associated with an increased risk of thrombocytopenia, hypocalcemia, hypernatremia, and leucopenia [13]. Serious complications can include cardiac arrest, NEC, sepsis, and neonatal death [8]. In some neonatal centers, hyperbilirubinemia in HDFN may also be treated with IVIG [3,9,11]. 

In the described case, phototherapy and IVIG were used to treat both twins. Exchange transfusion was contraindicated on the first day of life due to poor birth conditions, and was not necessary in the following days.

HDFN has been linked to cholestasis, as conjugated hyperbilirubinemia has been observed in 7–13% of neonates with HDFN. Recently, diagnostic criteria for cholestasis changed according to new guidelines (conjugated bilirubin concentration above 1 mg/dL instead of the previous standard), which might influence the prevalence of cholestasis diagnoses [3,4,5,9,14]. Hemolysis increases the concentration of free iron, which is stored mainly in the liver. In neonates with HDFN, ferritin levels are significantly above the normal range [15,16,17]. In both patients, ferritin concentrations exceeded the detection range of the test, reaching over 1650 ng/mL. On their 56th day of life, the twins were transferred to a multi-specialist hospital to continue differential diagnosis of cholestasis, where ferritin concentrations exceeding 5000 ng/mL were obtained from both patients. Iron overload may lead to liver dysfunction and cholestasis with coagulopathy, and severe cases may require chelation therapy [4,16,18,19,20,21,22]. 

The risk of cholestasis is higher in children who received IUTs and who have HDFN due to Rhesus D alloimmunization [3,4]. Higher risk is also observed in children with low birth weight, anemia, and high TSB and ferritin levels in the first days of life. Liver dysfunction may also be caused by hypoxia due to anemia and extramedullary hematopoiesis in the liver [4]. 

Our patients suffered from HDFN due to anti-D antibodies treated with IUTs, and hepatomegaly was observed. According to the literature, direct bilirubin concentrations of patients with HDFN can reach over 30 times the upper limit of normal [23]. In their first days of life, both twins developed severe cholestasis, with direct bilirubin exceeding 50 mg/dL in twin B. Similar cases of cholestasis since birth in patients with HDFN can be found in the literature. However, in no other case was such a high level (>50 mg/dL) of direct bilirubin described. In 1997, Grobler et al. reported the case of a term infant with HDFN complicated with kernicterus who had a maximal TSB concentration of 45.2 mg/dL, and a direct bilirubin of 31.6 mg/dL [23]. A 2022 review identified one patient with kernicterus due to hyperbilirubinemia caused by ABO hemolytic disease of the newborn. Their maximal TSB concentration reached 61 mg/dL, with a direct bilirubin of 27.7 mg/dL [24]. The author previously published a case report of a neonate with severe cholestasis and coagulation disorders in the course of hemolytic disease of the newborn who required chelation therapy, and who had a maximal direct bilirubin concentration of 33.14 mg/dL and a maximal ferritin concentration exceeding 33,000 ng/mL [22].

To our knowledge, the studies mentioned above identified the highest bilirubin levels reported in the available literature. 

The causes of cholestasis can be classified into two categories: extrahepatic, in which obstruction of biliary ducts occurs, and intrahepatic, in which dysfunction of hepatocytes or hypoplasia of intrahepatic biliary ducts is observed. Differential diagnoses must include biliary atresia, abdominal tumor, cholelithiasis, pancreaticobiliary anomalies, alpha-1 antitrypsin deficiency, galactosemia, bacterial infection, sepsis, listeriosis, viral infection (including TORCH), cystic fibrosis, Alagille syndrome, limy bile syndrome, long-term parenteral nutrition, toxic-induced and drug-induced liver injury, Caroli disease, and many others [6,25,26]. Necessary diagnostic imaging tests include abdominal ultrasound with evaluation of the bile ducts, hepatobiliary scintigraphy, liver biopsy, and cholangiography [6,25]. Laboratory tests that should be performed include tests for alpha-1 antitrypsin, the activity of GALT in RBCs, TSH, thyroid hormones, cortisol, electrolytes, and acid-base parameters, as well as an infection workup.

In our case, impairment of bile flow caused by HDFN and iron accumulation was deemed the most probable cause of cholestasis. Abnormal direct bilirubin levels had already been detected by the first day of life. The concentration of direct bilirubin could also have been influenced by other factors, e.g., NEC, parenteral nutrition, the severe condition of the children caused by HDFN, and the treatments used, such as antibiotic therapy.

Intrahepatic cholestasis requires causal treatment and diet therapy with partial replacement of long-chain triglycerides (LCT) for MCT, fat-soluble vitamin supplementation, and UDCA treatment [6,27].

Both patients received UDCA, vitamin supplementation, DHA with fish oil in parenteral nutrition, and high-MCT formula. No severe consequences of cholestasis were observed—the only symptoms present included pale stools and hepatosplenomegaly. Laboratory tests revealed low levels of vitamin D3, protein, and albumin, with normal values in the coagulation profile. 

According to the literature, bilirubin concentration in infants with HDFN normalizes within one week to three months. Laboratory results should be monitored until normalization [4]. In both patients, direct bilirubin slowly returned to the normal range. Currently, both twins are developing normally, require no medication, and receive outpatient care at a gastroenterology and hepatology clinic. The described patients do not present any signs of cerebral palsy or kernicterus.

## 4. Conclusions

This case report shows that HDFN may cause severe cholestasis in newborns. Patients with risk factors (such as an MCDA pregnancy, multiple IUTs, and poor birth condition) and extremal conjugated hyperbilirubinemia may be treated successfully with no severe consequential disability. It should be noted that long-term multidisciplinary care is necessary for these patients. Further investigation in this field is required to improve these outcomes.

This study, however, was subject to several limitations. First, a systematic search including additional databases may provide additional information on the subject. Also, some data from the patients’ medical histories might be missing due to multicenter management. Furthermore, the highest ferritin concentrations of the patients were unknown due to laboratory limitations.

## Figures and Tables

**Table 1:** Red blood cell parameters before and after intrauterine transfusions.

		TWIN A			TWIN B		
		Hgb (g/dL)	Hct (%)	RBC (1 × 10^12^/L)	Hgb (g/dL)	Hct (%)	RBC (1 × 10^12^/L)
IUT 1	Before	9.1	26.5	2.09	8.9	25.8	2.07
	After	12.4	35.5	3.36	13.1	38.0	3.69
IUT 2	Before	8.2	21.4	1.95	9.1	23.9	2.21
	After	12.6	33.8	3.51	12.8	35.1	3.55

IUT—intrauterine transfusion; Hgb—hemoglobin concentration; Hct—hematocrit; RBC—red blood cell count.

**Table 2:** Treatment details.

	Twin A	Twin B
Number of intrauterine transfusions	2 ^1^	2 ^1^
Number of top-up transfusions	3 ^2^	2 ^3^
Number of exchange transfusions	0	0
Duration of phototherapy in days	4	4
Number of intravenous immunoglobulin infusions	1 ^4^	1 ^4^
Days of parenteral nutrition	21	26
Days of parenteral nutrition without enteral feedings	8	12
Duration of antibiotic therapy in days	13	18
Length of stay in days	64 ^5^	64 ^5^

^1^ At 31 and 33 weeks of gestation. ^2^ In the 1st, 2nd, and 12th days of life. ^3^ In the 1st and 20th days of life. ^4^ In the 1st day of life. ^5^ Length of stay was defined as the time from birth until discharge from hospital to home. Both twins were treated in three hospitals.

**Table 3:** Initial and extreme laboratory results during hospitalization.

	Twin A	Twin B
Initial hemoglobin (g/dL)	8.1	9.7
Lowest hemoglobin (g/dL)	7.8	9.5
Initial total bilirubin (mg/dL)	6.7	9.9
Highest total bilirubin (mg/dL) (Age in days)	36.33 (7)	50.9 (18)
Total bilirubin at discharge (mg/dL)	1.99	1.99
Initial direct bilirubin (mg/dL)	1.94	5.51
Highest direct bilirubin (mg/dL) (Age in days)	32.21 (7)	50.2 (18)
Direct bilirubin at discharge (mg/dL)	1.87	1.88
Aspartate transaminase (AST) (U/L) (Age in days)	582 (14)	595 (29)
Alanine transaminase (ALT) (U/L) (Age in days)	607 (23)	366 (29)
Gamma-glutamyl transpeptidase (GGT) (U/L) (Age in days)	295 (19)	245 (29)
Lowest total protein (g/dL)	3.3	3.78
Lowest albumin (g/dL)	2.0	2.2
Initial ferritin (ng/mL)	932.5	>1650 *
Highest ferritin (ng/mL) (Age in days)	5281.76 * (57)	5839.65 * (57)

* The highest ferritin concentrations were obtained on the 57th day of life. Previous ferritin concentrations might have been higher, but due to laboratory limitations, they were labeled as above the detection range of the test (>1650 ng/mL).

## Data Availability

Data presented in this study are available in Table 1, Table 2 and Table 3 and Figure 1 and Figure 2.
